# Impact of Improved Design on Knudsen Force for Micro Gas Sensor

**DOI:** 10.3390/mi11070634

**Published:** 2020-06-28

**Authors:** Xiaowei Wang, Zhijun Zhang, Wenqing Zhang, Tianyi Su, Shiwei Zhang

**Affiliations:** School of Mechanical Engineering and Automation, Northeastern University, Shenyang 110819, China; xiaowwang812@163.com (X.W.); zhangwq1101@163.com (W.Z.); 1610113@stu.neu.edu.cn (T.S.); shwzhang@mail.neu.edu.cn (S.Z.)

**Keywords:** Knudsen thermal force, low-pressure gas sensor, direct simulation Monte Carlo (DSMC), MEMS

## Abstract

Knudsen force generated by thermally driven gas flow in a microscale structure has been used for gas detection and has shown immeasurable potential in the field of microelectromechanical system (MEMS) gas sensors due to its novel sensing characteristics. In this article, the performances of three kinds of Knudsen force gas sensors with improved isosceles triangular shuttle arm structures were studied. In the first design, the top side and right side lengths were equal; in the second, the top side and bottom side lengths were equal; and for the third, the bottom side and right side lengths were equal. A detailed investigation including gas flow, thermal characteristics, Knudsen force, and coupling effects between the shuttle-heater pairs was conducted using the direct simulation Monte Carlo (DSMC) method and the main mechanisms for gas flow presented were almost the same in this work. However, the second design returned the highest Knudsen force performance. The value increased by 42.9% (P = 387 Pa) compared to the Knudsen force of the original square shuttle arm. The results also demonstrate that the coupling effects become weak toward the right with an increase in the number of shuttle-heater pairs.

## 1. Introduction

In rarefied (low-pressure) gases without any initial pressure gradient, thermal stress is produced due to the inhomogeneous temperature. Thus, a gas flow is induced and exerts a force (Knudsen force or radiometric force) on the immersed structures. The effect first appeared in the famous Crookes radiometer developed by Sir William Crookes in the 1870s [1]. Ketsdever et al. [2] conducted a broad literature review for Knudsen force from the 19th to the 21st century. Note that the Knudsen force appears in the environment with a Knudsen number (Kn) higher than 0.1, which significantly limits its macroscopic application. Passian et al. [3,4] first demonstrated that the micro-cantilever focused by laser illumination can be deflected by the Knudsen force, fully displaying the application potential of Knudsen force at the micro and nano-scale.

With the advent of microelectromechanical system (MEMS) applications and the improvement in the relevant manufacture techniques, researchers and scholars have become more interested in Knudsen forces in recent years. Gimelshein and Ventura et al. [5,6] investigated Knudsen forces on a single vane in different sizes (thickness, height, and thickness-to-height ratio) and on a multi-vane with different gap-to-vane height ratios. The results demonstrated that (i) when the thickness-to-height ratio of the vane increased from 0.5 to 2, the Knudsen force decreased by 10–15%, thus a thinner vane is desirable for the device to operate efficiently; (ii) the Knudsen force on the sub-vanes is larger than that on the single vane with the same active area; and (iii) when the gap-to-vane height ratio is approximately 0.75, the Knudsen force on the vane reaches its maximum. Furthermore, it was found that if the edge geometry of the vane changed from straight into slanted, the overall Knudsen force on the vane will change. In particular, a vane with a sharp edge at the cold side helps to offset the decrease in Knudsen force with an increase in the vane thickness. For example, at the pressure of 1.2 Pa, when the vane thickness is increased from 0.5 cm to 1.5 cm, Knudsen forces on straight ($\alpha =90^{{}^{\circ}}$) and slanted ($\alpha =60^{{}^{\circ}}$) edged vanes decrease by 7.8% and increase by 5.9%, respectively [7]. Thus, vane configuration plays an important role in improving Knudsen force [7]. Strongrich et al. [8] analyzed the Knudsen force on a non-uniformly heated cantilever by combining the experimental measurement with numerical simulation. It was found that the Knudsen force is dependent on pressure, geometric shape, and temperature difference. Knudsen force coefficient is a nonlinear function of Kn, temperature ratio, and gap height to beam-width aspect ratio. It has been fully demonstrated that Knudsen force can be used in the actuation of micro-mechanical devices such as micro-sensors. Furthermore, the Knudsen force is highly favored in the fields of gas sensing and the measurement of pressure due to its great sensitivity to pressure, temperature, and gas composition [2,9,10].

**Figure 1:**
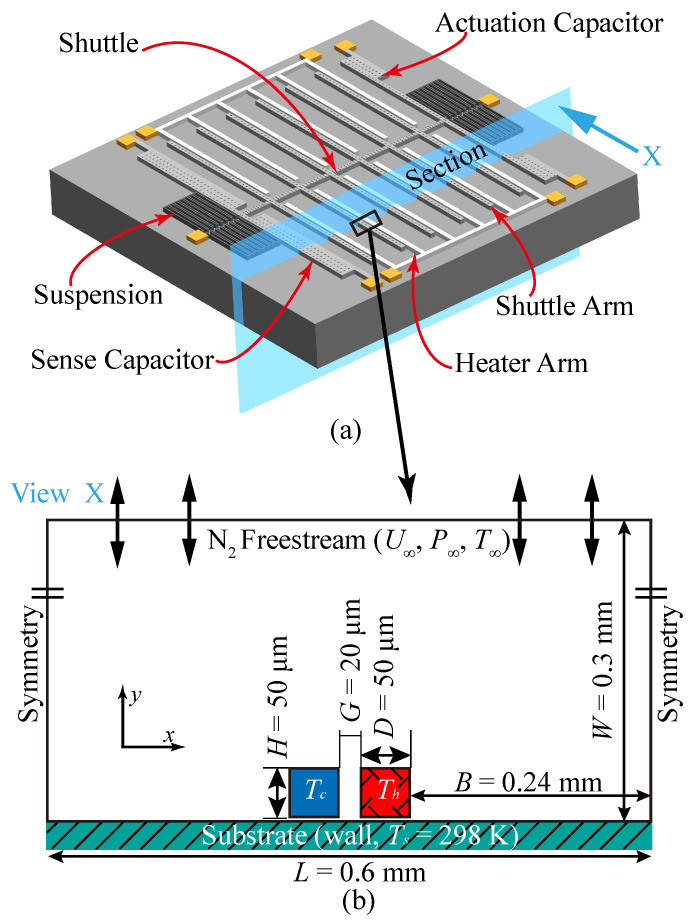
Schematics of the microelectromechanical in-plane Knudsen radiometric actuator (MIKRA) device and simulation domain. (**a**) MIKRA device [24], (**b**) MIKRA simulation model [25].

At present, we already have gas sensors such as gas chromatographs and mass spectrometers that can provide a very low limit detection, high accuracy, and selectivity. However, they have problems of large size, complicated structure, high price, and long response time [11,12]. There are commonly used chemiresistive, acoustic, optical, and capacitive gas sensors according to different working principles [13,14,15,16]. Chemiresistive gas sensors have advantages of easy fabrication, low cost, reuse ability, high stability, and sensitivity [17,18], however, the detecting properties are vulnerable to environmental factors such as humidity and temperature [17,18]. Generally, acoustic gas sensors have a high sensitivity and a short response time, but the detecting properties are easily affected by environmental temperature and sensing materials [19]. Optical gas sensors work on the basis of the changes of optical properties in the optical path length, scattering, absorbance, reflectivity, and refractive index [20]. This kind of sensor can work at room temperature and has advantages in high sensitivity, fast response, stability to environmental factors, and long lifetime [20,21]. Compared to the above three sensors, capacitive gas sensors have a higher sensitivity, selectivity, high-humidity instability, and low-temperature detecting properties [22,23]. Moreover, MEMS sensors have been widely researched and used thanks to their advantages of small volume, highly low power dissipation, easy mass production, low cost, and high sensitivity. A configuration variant of a micro gas sensor, namely the microelectromechanical in-plane Knudsen radiometric actuator (MIKRA) has recently been reported [24], as shown in Figure 1a. MIKRA is composed of two micro-beam arrays: one consists of movable shuttle arms and the other includes the fixed heater arms; these are separated by a gap to the order of 20 μm. The shuttle is linked with a serpentine spring element, suspended 4 μm above the substrate. The shuttle itself consists of 12 arms extending transversely outward. The heater arms that are the same size as the shuttle arm are anchored to the substrate using thermal oxide. The heater arms contain a platinum heating element heated by a proportional-integral (PI) controller. The temperature of the heater arms can be controlled by adjusting the output power (75, 100 and 125 mW) of the PI controller, and it rises through exerting an electric current, producing a temperature gradient in the gap on the order of 10^6^ K/m [25]. Thus, the Knudsen force appears and drives the shuttle arm to deviate from the heater arm. The displacement of the shuttle arm is measured by the comb capacitor at one end of the shuttle. In the experiment, a mass flow controller was used to assure that the device was in the gas mixture with a specified concentration and we increased the temperature of the heater arms to make the shuttle arms deviate from the heater ones, and then the magnitude of this deflection was measured. A functional relationship between the magnitude of deflection and the concentration was constructed by repeating the above two operations, so as to calibrate the corresponding gas concentration. To analyze the flow field characteristics in the device, the authors constructed a two-dimensional (2D) domain model, as shown in Figure 1b [25]. Later, Gerdroodbary et al. [26,27,28,29,30,31,32,33] conducted a lot of research and found that the gas flow was induced by (nonlinear) thermal stress flow, thermal creep flow, and thermal edge flow. Among these three gas flows, the (nonlinear) thermal stress flow plays the main role.

Compared to the micro-cantilever structure [10], the MIKRA device has a higher Knudsen force coefficient, and the peak value increases approximately seven times [24]. In order to analyze how the working condition and geometric parameter help to improve MIKRA performance, Gerdroodbary et al. [26] found that the peak value of Knudsen force will increase more than seven times if the temperature difference between the shuttle and heater arms is increased from 10 K to 100 K. Moreover, through increasing the arm height and height-to-thickness ratio, they found that [27]: (i) the Knudsen force has a peak value enhancement of about 3.66 times by increasing the arm height to three times; and (ii) the peak value of the Knudsen force will increase about 5.33 times if the height-to-thickness ratio is increased nine times. Recently, Zheng et al. [34] also demonstrated that the increase in arm height could improve the Knudsen force by applying response surface methodology (RSM). Meanwhile, they pointed out that both an increasing arm gap and decreasing operating pressure could help to improve Knudsen force. Note that Knudsen force will not keep increasing with the increase of pressure because Knudsen force, a function of pressure, has the characteristic of a bell-shaped distribution.

On the other hand, within an ambient pressure of 25 Pa to 1500 Pa, MIKRA can achieve the detection of gases (N_2_–H_2_O, Kr–Xe, He–Ne–Ar, N_2_–H_2_, CH_4_–SO_2_, CO_2_, N_2_–NH_3_, and Air–H_2_) [28,29,30,31,32,33,34,35,36,37]. Knudsen force is linearly dependent on the concentration of any gas species in a gas mixture [36]. Li et al. [37] employed RSM to obtain the correlations between Knudsen force and the factors (pressure, temperature difference between the shuttle and heater arms, gap size, and mass fraction of the hydrogen in the gas mixture) in MIKRA devices. The results demonstrate that the efficiency and precision of MIKRA devices improved significantly as the mass fraction of hydrogen in the gas mixture increased.

To analyze the interactions between the shuttle-heater pairs and coupling effects including flow field, thermal characteristics, and Knudsen force, Pikus et al. [38] constructed a MIKRA model consisting of three shuttle-heater pairs and applied the research method of direct simulation Monte Carlo (DSMC). The results demonstrated that the force on the leftmost shuttle arm was the largest; the forces on the middle and rightmost shuttle arms were approximately the same and equal to the experimental values. Furthermore, the streamlines in the simulation domain presented coupling effects, suggesting that the simulation work could accurately represent the experiment by doing more work with boundary conditions [38].

Based on the research backgrounds above-mentioned, alternative designs for MIKRA devices with enhanced Knudsen force performance were proposed and assessed in this paper. The results were compared with the experimental and numerical data of Strongrich et al. [24,25]. Additionally, the influence of the boundary condition and simulation domain size on the interactions between the shuttle-heater pairs was also compared. The remainder of this paper is organized as follows. In Section 2, the varieties of model configurations and geometric parameters are detailed. A brief description of the DSMC method applied in this work is given and the corresponding boundary conditions and simulation parameters are confirmed in Section 3. In Section 4, the DSMC simulation results of the flow field, thermal field, Knudsen force, and coupling effects characteristics are presented. In Section 5, our conclusions and suggestions are given.

## 2. Problem Statement

**Figure 2:**
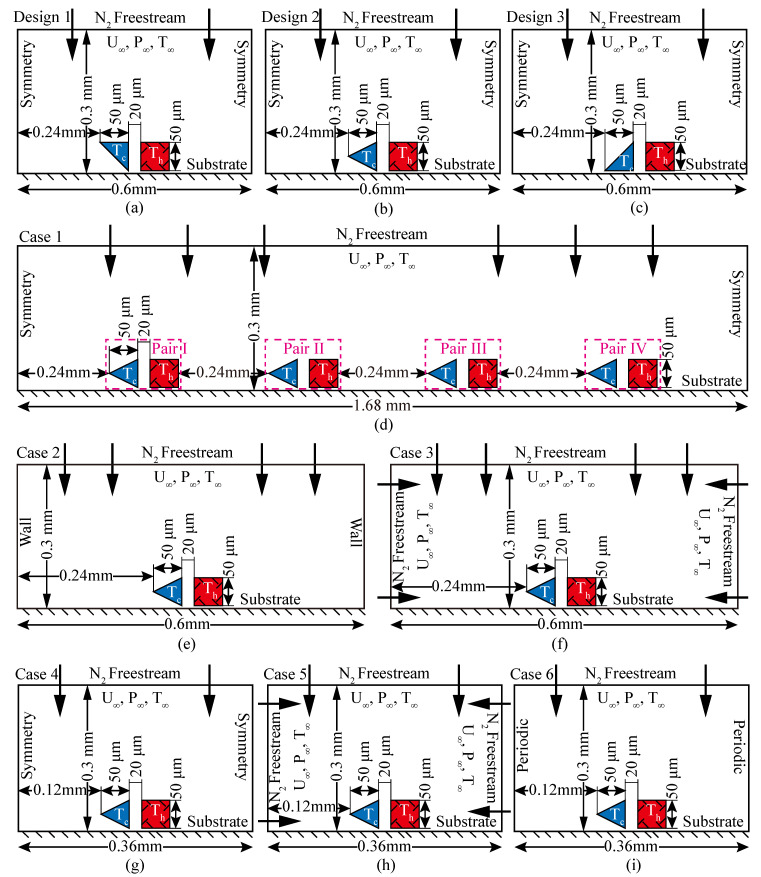
Analyzed configurations and geometric parameters. (**a**–**c**) Three geometric configuration designs, (**d**) coupling effects configuration case, (**e**–**i**) five boundary condition configuration cases.

Here, three kinds of shuttle arm designs were investigated to enhance the Knudsen force, as shown in Figure 2a–c. The shuttle arm structure of design 1 was an isosceles right triangle (top side length equals right side length), as shown in Figure 2a; that of design 2 was an isosceles triangle (top side length equals bottom side length), presented as Figure 2b; and that of design 3 was an isosceles right triangle (bottom side length equals right side length), as shown in Figure 2c. Compared with the original MIKRA design (Figure 1b), these three designs only had a different geometric shape. That is, the shape of the shuttle arm changed from square to triangular. All of the other parameters remained the same.

To further analyze the coupling effects, a model with a larger simulation domain including four shuttle-heater pairs was constructed based on design 2 (the reasons for selecting design 2 are discussed in the next section), as shown in Figure 2d (case 1). The four shuttle-heater pairs were pair I, pair II, pair II, and pair IV in the order from left to right. In this model, all the shuttle and heater arms had the same temperature to ensure that the temperature difference between the shuttle and heater arms of each pair was equal. The distance between the shuttle-heater pairs was 240 μm and the size of the whole simulation domain was 1680 μm × 300 μm.

Furthermore, to investigate the influence of the boundary condition and simulation domain size on the simulation results and coupling effects, five cases (Figure 2e–i) were assessed. Cases 2 and 3 were obtained by replacing the symmetry boundary condition (left and right boundaries) of design 2 with the wall (Figure 2e) and freestream (Figure 2f) boundary conditions, respectively. In case 4 (Figure 2g), the left and right boundaries were symmetry boundaries, and the simulation domain size was 0.36 mm × 0.3 mm, obtained by shrinking half of the distance between the shuttle arm (heater arm) and left (right) boundary in the x direction of design 2. Shrinking the distance decreases the simulation domain size, total number of cells, and overall simulation particles, helping to shorten the computational time. Cases 5 and 6 had the same sizes as case 4, but were configured as freestream (Figure 2h) and periodic (Figure 2i) boundary conditions, respectively. Specifically, when gases collide with a wall, gas-surface interaction effects, specular or diffuse reflection, will occur. However, this will not happen for the freestream and periodic boundaries. When colliding with the freestream boundary, the gas is considered to leave the flow field. For the periodic boundary, gases flowing out of the simulation domain from one side will flow into it from the other side. The geometric parameters and temperature difference of the shuttle-heater pair remained unchanged in all of the designs, which was convenient for the comparison of DSMC simulation results.

## 3. Numerical Approach

### 3.1. Direct Simulation Monte Carlo (DSMC) Method and Solver

DSMC [39], a stochastic particle-based method, is used to simulate the gas flow in an actuator. The computational fluid dynamics (CFD) method, based on the classic Navier–Stokes equation, is no longer applicable due to a large Kn. Instead, the Boltzmann equation is needed. DSMC is a highly-efficient method to solve the Boltzmann equation [40,41]. Moreover, the DSMC method has been widely used in the research of rarefied gas flow induced by temperature field [42,43,44] and low-pressure gas actuators based on Knudsen force [25,26,27,28,29,30,31,32,33,34,35,36].

DSMC simulates the rarefied gas flow by using a large number of representative simulated particles to reproduce the behavior of real gas. Every simulated particle represents a great number of real gas molecules. Information of the location, velocity, and internal energy of each particle would be saved in the computer and changed with its motion, collision, and interaction with the walls. The simulated particles in the grid are tracked, sampled, and then averaged to achieve the macroscopic properties. An open source DSMC solver, dsmcFoamPlus [45], was applied in this work, and its low version, dsmcFoam [46], has already been used in the research of MIKRA [26,27,28,29,30,31,32,33]. Variable hard sphere (VHS) and variable soft sphere (VSS) are common models to describe the collisions between particles. Generally, the VHS model is used for matters of single rarefied gas flow while the VSS model is for gas mixture flows. In this work, a single gas, N_2_, was considered, thus the VHS model was applied to the collisions between the simulated particles. In addition, the Larsen–Borgnakke (LB) model [47] was employed because N_2_ is a diatomic molecule and the exchange of translational energy and internal energy occurs in the process of collisions between particles. Based on the standard no time counter (NTC) collision method [39], collision pairs were selected. The NTC scheme considers a maximum number of collision rate per time interval (Δt), which determines the number of randomly selected particle pairs that should be checked for potential collisions.

### 3.2. Parameter and Boundary Condition

2D simulation was considered. In this paper, the pressure ranged from 62 Pa to 1500 Pa. Beyond this pressure range, the value of Knudsen force was negative, which means that the shuttle arm is drawn by the heater arm, which is not expected. The temperatures of the shuttle and heater arms for different pressures are listed in Table 1 [25,30]. These temperatures were obtained based on the average values of the experimental data [24,25] in pure nitrogen gas. All simulations were operated in a N_2_ ($m=46.5\times 10^{-27}\;\mathrm{kg},\;d=4.17\times 10^{-10}\;m$) environment to compare the results with other simulation and experimental data [24,25,30].

The simulation domain was discretized and the maximum cell dimension (Δx=Δy=2 μm) was obtained. Due to the application of the NTC method, the number of simulated particles within each cell was no less than 20 for the initialization [26,27,28,29,30,31,32]. Δt, similar to the previous work [25], was smaller than the minimum values of the cellular transit time and mean collision time (i.e., $\Delta t=1\times 10^{-9}\;s$). To reduce the statistical errors of the DSMC data and achieve a steady condition of the result, all of the simulations involved at least $3\times 10^{6}$ time intervals. Table 2 presents some detailed information such as cell size, particles per cell (PPC), time interval, number of time intervals, and the total number of cells. The total number of cells for each simulation case was different due to different simulation domain sizes.

The boundaries of the shuttle arms, heater arms, and substrates in all of the simulations were set as isothermal walls. The collision processes of the gas molecules with these wall surfaces are described by the well-known diffuse gas-surface interaction model (i.e., the gas molecules reach thermal equilibrium with the surface temperature in the process of collision). The top boundaries with the freestream condition were used to simulate the flow-in and flow-out of N_2_. The left and right boundary conditions (symmetry, isothermal wall, freestream, and periodic) were dependent on the simulation cases (Figure 2a–i).

## 4. Results and Discussion

### 4.1. Flow Mechanism and Patterns

Previous studies [26,27,28,29,30,31,32,33] have already briefly analyzed the gas flow mechanism in MIKRA devices. Here, the flow mechanism was investigated in detail and the influence of the geometric designs is also discussed. The flow mechanisms were similar in all geometric designs in the current work.

**Figure 3:**
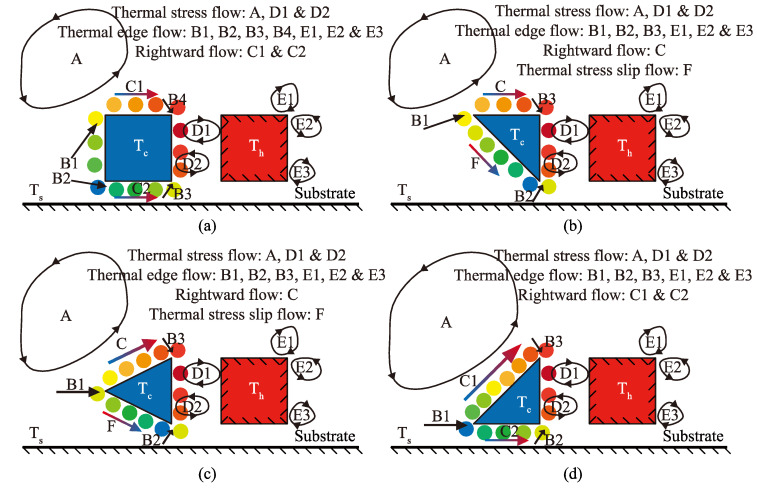
Schematics of flow mechanism. (**a**) MIKRA original design, (**b**) improved design 1, (**c**) improved design 2, (**d**) improved design 3. Circular symbols depict gas particles, and their colors indicate gas temperature.

Gas temperature gradient (for the quantitative information, refer to Section 4.2) and thermally driven flow along the shuttle arm surfaces are illustrated as Figure 3. (Nonlinear) thermal stress flow is induced by stress due to the non-uniform temperature distribution [44,48], thus, three circulation flows or vortices (flow A, flow D1, and flow D2 in Figure 3) were formed. Flow A had the largest volume and was the main circulation flow. Flows D1 and D2, flowing in the opposite direction, occurred in the gap between the shuttle and heater arms. Furthermore, the sharp edges of the configuration (shuttle and heater arms) structure resulted in a dramatic temperature change around the tips. Thus, thermal edge flows appeared in the vicinity of the tips [48], shown as flows B1–B4 and flows E1–E3 in Figure 3. Thermal edge flow is the main reason why the pressure difference occurs in the vicinity of the configuration edges. The location and number of the thermal edge flows differed for the different shuttle arm structures. For example, the number of thermal edge flows near the tips of the shuttle arm in the original MIKRA design was four (B1-B4), as shown in Figure 3a; but that of designs 1–3 was three (B1-B3), as shown in Figure 3b,c.

Gas temperature gradient occurred along both the top surface and bottom surface of the shuttle arm, thus gas flows in the right direction (flow C/C1/C2 and flow F) were produced, as shown as colorful arrows in Figure 3. For all designs, the gas temperatures over the top surface increased gradually from left to right, thus, rightward flows C/C1 were observed [43]. Note that the rightward flow was similar to the thermal creep flow; both were from the cold side to the hot side. However, the thermal creep flow appeared along the wall surface with temperature gradient [48,49,50], while the rightward flow was along the constant temperature surface. Over the bottom surfaces, gas temperatures, compared to the value of pair I, increased gradually from left to right in the original design and design 3, thus, rightward flow C2 was produced, as shown in Figure 3a,d. However, in designs 1 and 2, the gas temperatures over the bottom surfaces decreased gradually from left to right, thus thermal stress slip flow F was induced [48], flowing from the high temperature region to low temperature region, as shown in Figure 3b,c. The strength of the gas flows above varied due to different pressures and gas species [2,26,27,28,29,30,31,32,33,34,35,43,44]. Additionally, the mass concentration of the gas was considered in the gas mixture [26,27,28,29,30,31,32,33,34,35].

**Figure 4:**
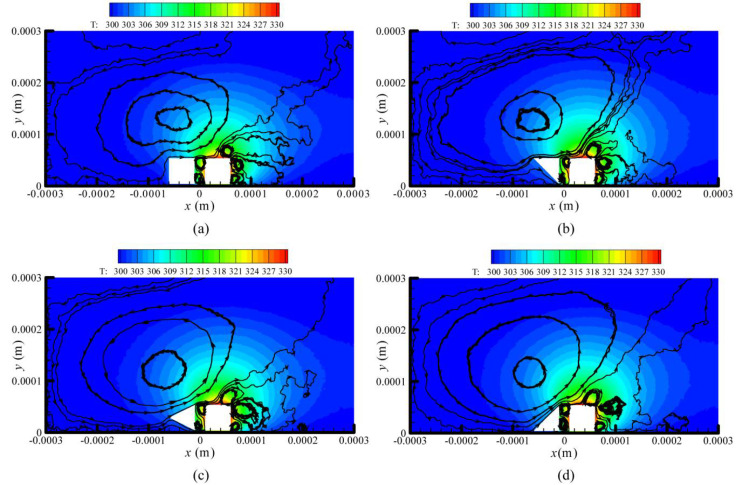
Streamlines and temperature contours of the four designs for P = 387 Pa. (**a**) MIKRA original design [35], (**b**) improved design 1, (**c**) improved design 2, (**d**) improved design 3.

The streamlines and temperature contours of the four designs for P = 387 Pa are presented in Figure 4 and shows that the gas flow patterns were very similar. The variation of the shuttle arm configuration did not affect the flow patterns of the thermal stress flow (flows A, D1, and D2) and thermal edge flow (flows E1, E2, and E3) near the sharp edges of the heater arm. These similar flow patterns have also been observed in previous research [26,27,28,29,30,31,32,33,34,35].

However, the flow pattern around the vicinity of the shuttle arm was significantly influenced by the shuttle arm geometric design. Due to the thermal stress slip flow, design 1 had more gases flow by the bottom surface than the original design, as shown in Figure 4b. By comparing designs 1–3, it can be found that the thermal stress slip flow over the bottom surface becomes weak gradually, thus, fewer gases flow by it. In contrast, the rightward flow over the top surface becomes strong gradually, with more and more gases flowing by it. Note that the gas flows over the bottom surface would pass through the gap between the shuttle and heater arms. On the other hand, in the original design, gas flow in the right direction is blocked by the left surface of the shuttle arm. Regarding designs 1 and 2, the bottom surfaces block the gas flows, and design 2 blocked them less. The top surface of design 2 was not found to block the gas flows, but in design 3, the top surface blocked the gas flows, but blocked them less than the bottom surface of design 1. Thus, it was presumed that design 2 returned the best performance.

**Figure 5:**
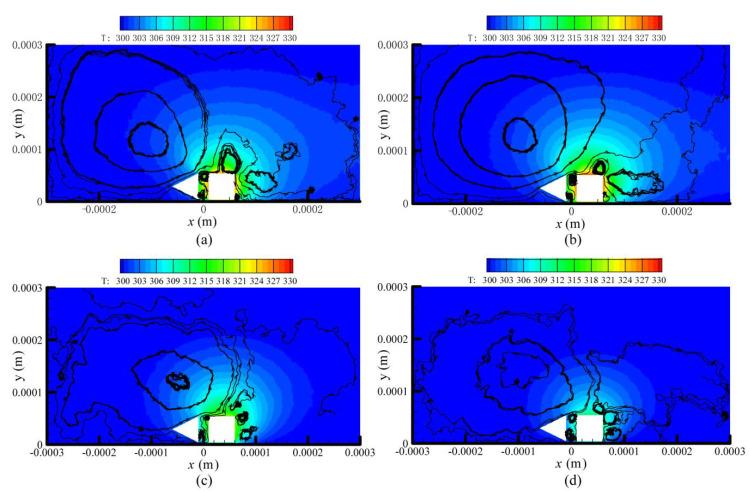
Streamlines and temperature contours of design 2 for different values of pressure. (**a**) P = 62 Pa, (**b**) P = 155 Pa, (**c**) P = 966 Pa, (**d**) P = 1500 Pa.

Figure 5 shows the flow patterns and temperature distributions of design 2 at different pressures. The flow patterns did not change significantly as the pressure increased, but the streamlines became fluctuant. In addition, the volume of the main vortex A had a trend of increasing first and then decreasing. The center of vortex A gradually moved toward the right as the pressure increased with P ≤ 966 Pa. If the pressure continues to increase, the center of vortex A will move away from the heater arm. The qualitative variation of gas flow pattern for different pressures was observed in the previous research of the original design [28,29,30,35,36].

### 4.2. Temperature and Heat Transfer

**Figure 6:**
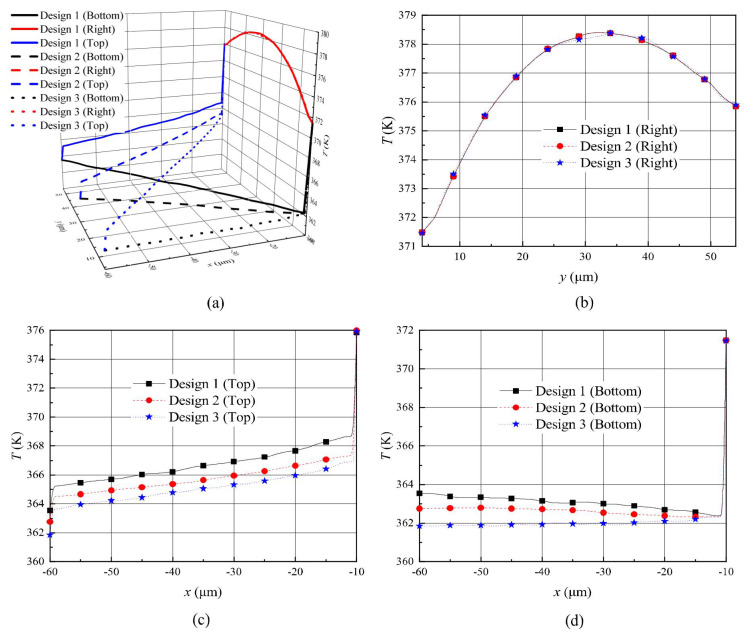
Temperature jump distributions over the shuttle arm surfaces for different designs with P = 387 Pa. (**a**) All shuttle arm surfaces, (**b**) right surface, (**c**) top surface, (**d**) bottom surface.

The temperature distributions showed some similarity when comparing the temperature contours of previous reports [26,27,28,29,30,31,32,33,34,35] and Figure 4. Temperatures of all wall boundaries were constant, but due to the rarified gas effects, a temperature jump and velocity slip appeared near the wall surfaces. In order to further analyze the influence of different designs on the gas temperature over the shuttle arm surface quantitatively, the gas temperature jump distribution over the surfaces is presented, as shown in Figure 6. The gas temperatures over all surfaces are presented in Figure 6a. The detailed temperature variations in the y direction and x direction are shown in Figure 6b–d.

For designs 1–3, the temperature distributions over the right surfaces were similar. The temperatures increased first and then decreased with the increase in the shuttle arm height ($y=4\times 10^{-6}\to 5.4\times 10^{-5}$), which was similar to that reported in [25,35]. This is because the lowest part of the shuttle arm was close to the substrate, thus having the lowest temperature. Affected by the heater arm, the temperature rose gradually as y increased within some range. If y continues to increase to the highest part of shuttle arm, the temperature affected by the cold gas will decrease. Furthermore, their temperature values were almost the same, as shown in Figure 6a,b. The gas temperature over the top surface increased gradually with a decrease in the shuttle arm width ($x\;=\;-6\times 10^{-5}\;\to \;-1\times 10^{-5}$), which was similar to that in [25]. This is because the temperature over the top surface was significantly influenced by the heater arm on the ride side. The top surface of design 1 had the shortest distance from the heater arm, thus its temperature was the highest, but that of design 3 was farthest from the heater arm, thus had the lowest temperature. Design 2 had a temperature over top surface ranging between designs 1 and 3. Additionally, for all designs, the temperature curves had two jumps at the junctions of the top-bottom surface and the top-right surface, as shown in Figure 6c. Additionally, a temperature jump over the bottom surfaces could be observed. That is, the temperature curves had one jump at the junction of the bottom-right surface, as shown in Figure 6d.

As expected, a dramatic temperature discontinuity was produced at the junction. This fully demonstrates that thermal edge flow occurred at the edges of the shuttle arm surfaces as analyzed above. The temperature distributions over the bottom surfaces of designs 1–3 were not exactly the same. The gas temperature over the bottom surfaces of designs 1 and 2 decreased gradually, while that of design 3 increased slightly with the decrease in the shuttle arm width, which was similar to that reported in [25,35]. The reason for this is that the bottom surfaces of design 1 and 2 got closer and closer to the substrate with the shuttle arm width decreases (be closer to the heater arm), but that of design 3 had an unchanged distance from the substrate. Obviously, the minimum temperature of the bottom surface corresponded to design 3, as shown in Figure 6d, as the temperature of the bottom surface was more easily influenced by the substrate. The bottom surface of design 1 was farthest from the substrate, thus, had highest temperature. Similarly, design 2 had a bottom surface temperature between designs 1 and 3.

**Figure 7:**
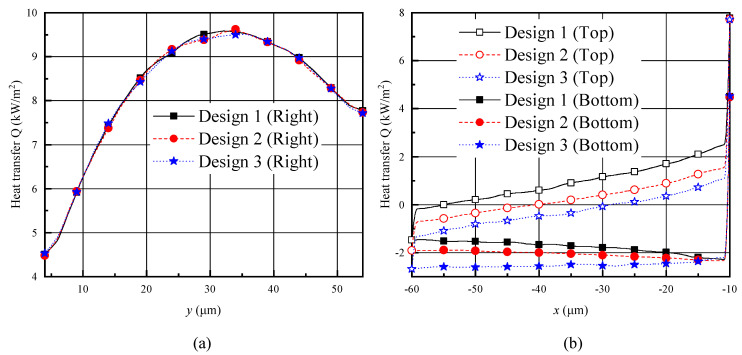
Heat transfer distributions over the shuttle arm surfaces for different designs with P = 387 Pa. (**a**) Right surface, (**b**) top and bottom surfaces.

In order to better understand the gas temperature distributions over the shuttle arm surfaces, the heat transfer distributions are illustrated in Figure 7. Positive heat transfer indicates the shuttle arm is absorbing heat while the negative one is releasing heat. The heat transfer distributions are similar to the temperature distributions [25,35]. The right surfaces of the shuttle arms are closer to the heater arms, thus absorbing more heat [25,35]. The heat transfers over the right surfaces in designs 1–3 were almost the same, as shown in Figure 7a. As expected, the top and bottom surfaces of design 1 absorbed the most heat and released the least heat, respectively. However, the top and bottom surfaces of design 3 absorbed the least heat and released the most heat, respectively, as shown in Figure 7b. Design 2 ranged between designs 1 and 3. This kind of heat transfer also resulted from different distances between the shuttle arm surfaces (bottom and top) and the substrate and heater arm, as analyzed in the above two paragraphs.

### 4.3. Pressure Distribution and Knudsen Thermal Force

**Figure 8:**
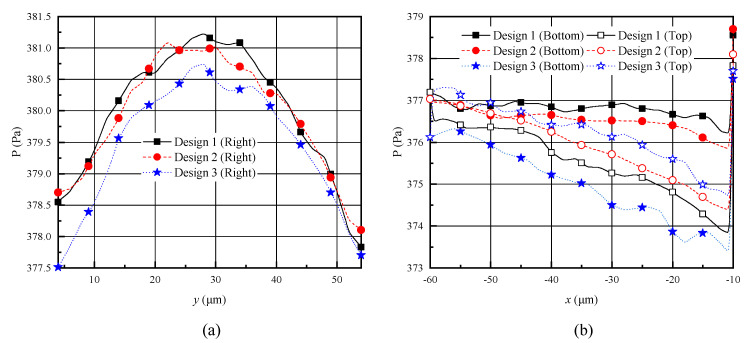
Pressure distributions over the shuttle arm surfaces for different designs with P = 387 Pa. (**a**) right surface, (**b**) top and bottom surfaces.

Figure 8 illustrates the pressure distributions over the shuttle arm surfaces for analyzing the Knudsen force. As reported in previous research [25,26,27,29,35], (i) the pressure on the right side is larger than that on the top and bottom sides, and (ii) the highest pressure is at the center of the right side. This means that the shuttle arm is repulsed from the heater arm. No obvious difference of the pressure distribution was observed for different geometric designs of the shuttle arm, but the pressure values were different. The difference of the pressure value might result from the different gas flow patterns. As discussed above, among the improved designs 1–3, design 1 had the most gases flow by the bottom surface and the gap between the shuttle and heater arms; design 2 had less; and design 3 had the least, where almost all gases flowed by the top surface. Therefore, the pressure on the right and bottom surfaces of design 1 was the highest, but that of design 3 was the lowest. However, the pressure on the top surface of design 3 was the highest, while that of design 1 was the lowest. In terms of design 2, the pressure value ranged between the values of design 1 and design 3. As the Knudsen force is mainly decided by the pressure difference between the two sides of the shuttle arm, pressure variation due to different geometric designs directly affects the Knudsen force.

**Figure 9:**
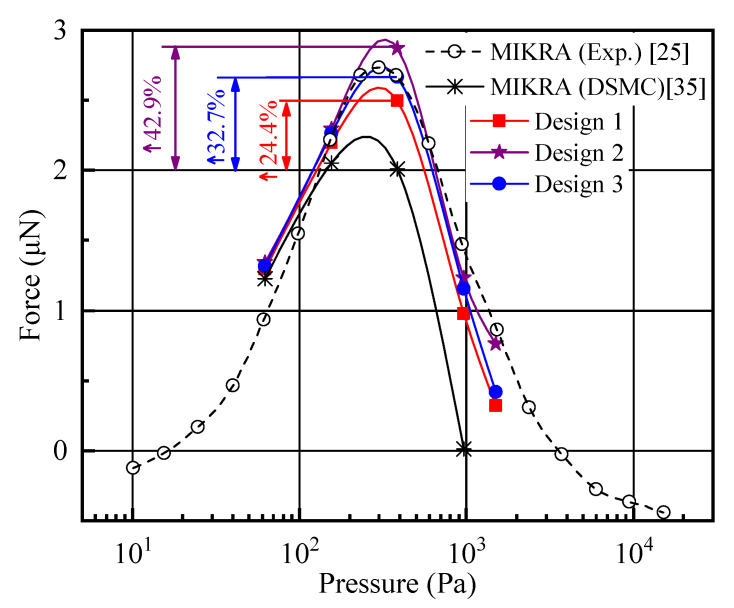
Knudsen forces (twelve pairs) for the different designs.

From the above analysis, it can be found that the gas temperature jump, heat transfer, and pressure value over the surfaces of design 2 ranged between that of designs 1 and 3, without presenting the advantage of design 2. In fact, the Knudsen force is an index of the sensing property of the MIKRA device. The Knudsen forces (twelve pairs) of three shuttle arm designs were compared with the previous DSMC numerical [35] and experimental [24,25] data of the original MIKRA device, as shown in Figure 9. The results displayed a good qualitative agreement. The Knudsen force distributions over the shuttle arm for different designs had a bell-shaped characteristic with the increase in pressure [24,25,26,27,28,29,30,31,32,33,34,35]. Obviously, the influence of the geometric design on the Knudsen force was significant. The DSMC numerical result of original MIKRA design was the smallest among the DSMC simulation data. In particular, for the three designs considered, design 2 returned the largest Knudsen force, and design 3 returned the second largest. Compared to the DSMC numerical results of the MIKRA device, designs 1–3 returned Knudsen forces increased approximately by 24.4%, 42.9%, and 32.7%, respectively, with P = 387 Pa. On the other hand, compared to the experimental data of the MIKRA device, design 3 returned the approximate Knudsen force. However, design 1 and design 2 returned Knudsen forces approximately decreased by 6.3% and increased by 7.7%, respectively, with P = 387 Pa. The reasons for the difference between the DSMC numerical and experimental data were believed to be the 2D domain, the constant arm temperature, and the fixed distance between the shuttle and heater arms during the simulation process [25,26,27,28,29,30,31,32]. However, these influence factors were mainly replaced by the geometric configurations of the shuttle arm in the current work.

### 4.4. Coupling Effects

Design 2 was used to investigate the coupling effects since it had the best Knudsen force performance from the above discussion. Moreover, design 2 was applied to analyze the influence of the boundary conditions (wall, freestream, and periodic) and simulation domain sizes (0.6 mm × 0.3 mm, and 0.36 mm × 0.3 mm) on the device performance. The parameter information matched that of cases for Kn = 0.742.

**Figure 10:**
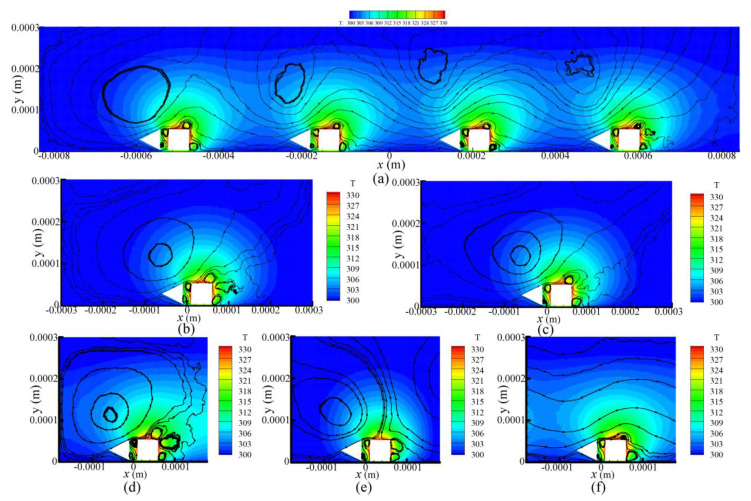
Streamlines and temperature contours at P = 387 Pa for different boundary conditions and domain sizes. (**a**) Four heater-shuttle pairs, (**b**) wall boundary condition, (**c**) freestream boundary condition, domain size of 0.6 mm × 0.3 mm, (**d**) symmetry boundary condition, (**e**) freestream boundary condition, domain size of 0.36 mm × 0.3 mm, (**f**) periodic boundary condition.

Streamlines and temperature contours of the cases are shown in Figure 10. The coupling effects could be found through the streamlines in the flow field of case 1, as shown in Figure 10a. It was also found that (i) the volume of the main vortex A shrank from left to right, and (ii) vortices E1 and E3 appeared near the heater arm tips of four shuttle-heater pairs, but vortex E2 only occurred near the heater arm tip of pair IV (in the rightmost side). These phenomena were also observed in the previous work [38], but more gases flowed by the bottom surface of the shuttle arm and the gap between the shuttle and heater arms in the current work due to the different geometric design. The flow fields of one-pair (Figure 4c) and four-pairs were similar, which means that independence exists between the shuttle-heater pairs. Therefore, the gas flow behavior and performance of the MIKRA device can be simulated by only one shuttle-heater pair.

The flow patterns of cases 2–4 were highly similar, which indicates that more research work can be done in terms of the boundary condition and domain size to improve the simulation efficiency and to accurately represent the experiment [38]. On the other hand, in cases 5 and 6, a large amount of gases flowed in or flowed out of the left and right boundaries since the boundary conditions were respectively set as freestream and periodic. The flow patterns of case 5 did not agree with the actual situation (Figure 10a). The reason seems to be that the combination of the boundary condition and simulation domain size in case 5 could not simulate the gas flow behavior of the MIKRA device. Regarding case 6, it can be considered as an ideal situation including countless shuttle-heater pairs. Since 2D simulations were implemented in the current work, the mass flow rate was calculated with a width of 1 μm in the z direction. The mass flow rate of 1.33 × 10^−9^ kg/s was obtained over a vertical line connecting the substrate to the top freestream. Thus, case 6 demonstrates that micro-configuration has potential in the application of gas pumping such as the Knudsen pump (compressor) [51,52,53,54].

**Figure 11:**
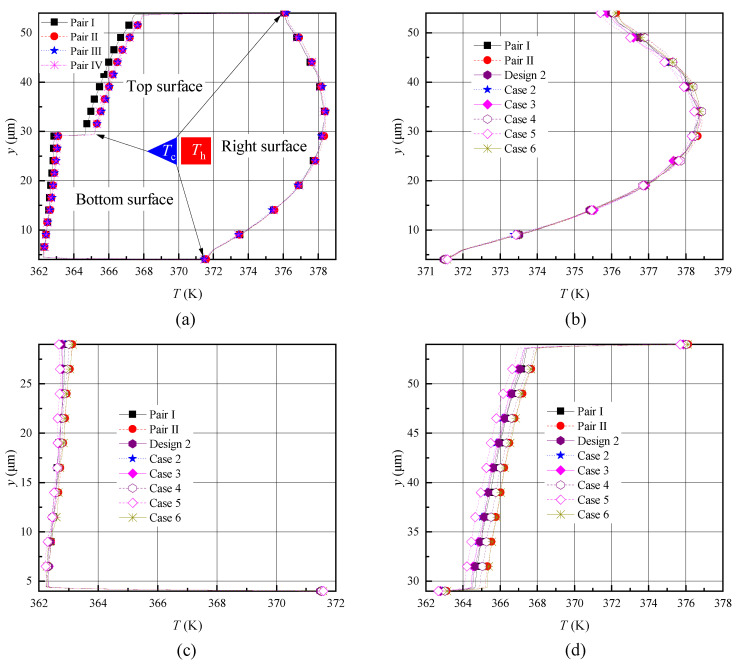
Comparison of the temperature jump distributions over the shuttle arm surfaces for different boundary conditions and domain sizes with P = 387 Pa. (**a**) All surfaces for four heater-shuttle pairs, (**b**) right surface, (**c**) bottom surface, (**d**) top surface.

Regarding the temperature contours (refer to Figure 10), some differences for different boundary conditions and domain sizes were observed. The quantitative information of the temperature jump distribution over the shuttle arm surfaces to illustrate the difference is given in Figure 11. Obviously, in the model with four shuttle-heater pairs (case 1), the rules of temperature distribution over all surfaces were similar. More specifically, the temperatures over the right surfaces were almost the same, but both of the temperatures over the top and bottom surfaces of pair I were slightly lower than that of the other three pairs (pairs II, III, and IV). Moreover, the temperatures over the top and bottom surfaces for those three pairs (pairs II, III, and IV) were almost equal, as shown in Figure 11a.

**Figure 12:**
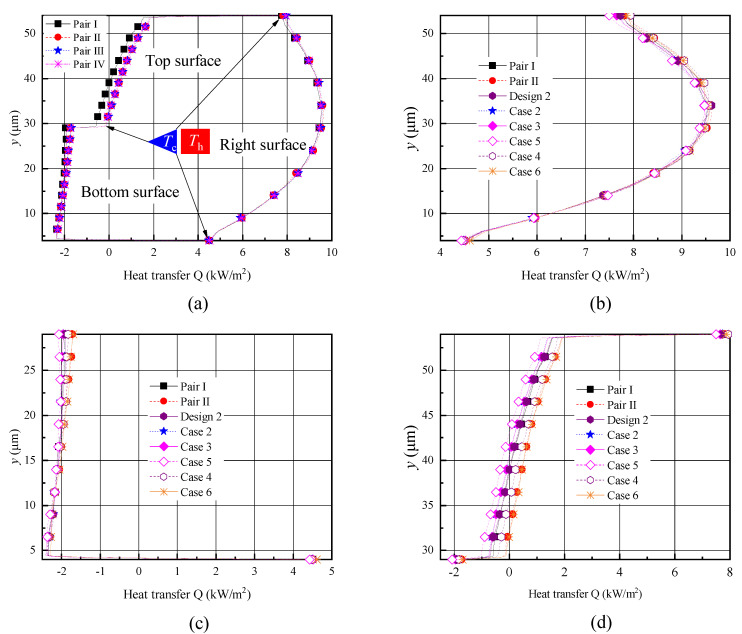
Comparison of heat transfer distributions over the shuttle arm surfaces for different boundary conditions and domain sizes with P = 387 Pa. (**a**) All surfaces for four heater-shuttle pairs, (**b**) right surface, (**c**) bottom surface, (**d**) top surface.

By comparing the data of pairs I and II in case 1 and cases 2–6, it was found that the temperatures over the right surfaces were similar, as shown in Figure 11b. In terms of the temperatures over the bottom and top surfaces as presented in Figure 11c,d, it can be seen that (i) the temperature of pair II was almost the same as that of case 6 (Figure 2i) and this value was the highest in all cases, however, the temperature of case 5 was the lowest (Figure 2h); and (ii) the temperatures of pair I and cases 2–4 were close, ranging between the above two values of cases 5 and 6. Note that the heat transfer distributions over the shuttle arm surfaces had a similar situation (Figure 12), since heat transfer is closely related to temperature [25,35]. That is, absorbing more (releasing less) heat results in a higher temperature, otherwise it will be a lower temperature.

**Figure 13:**
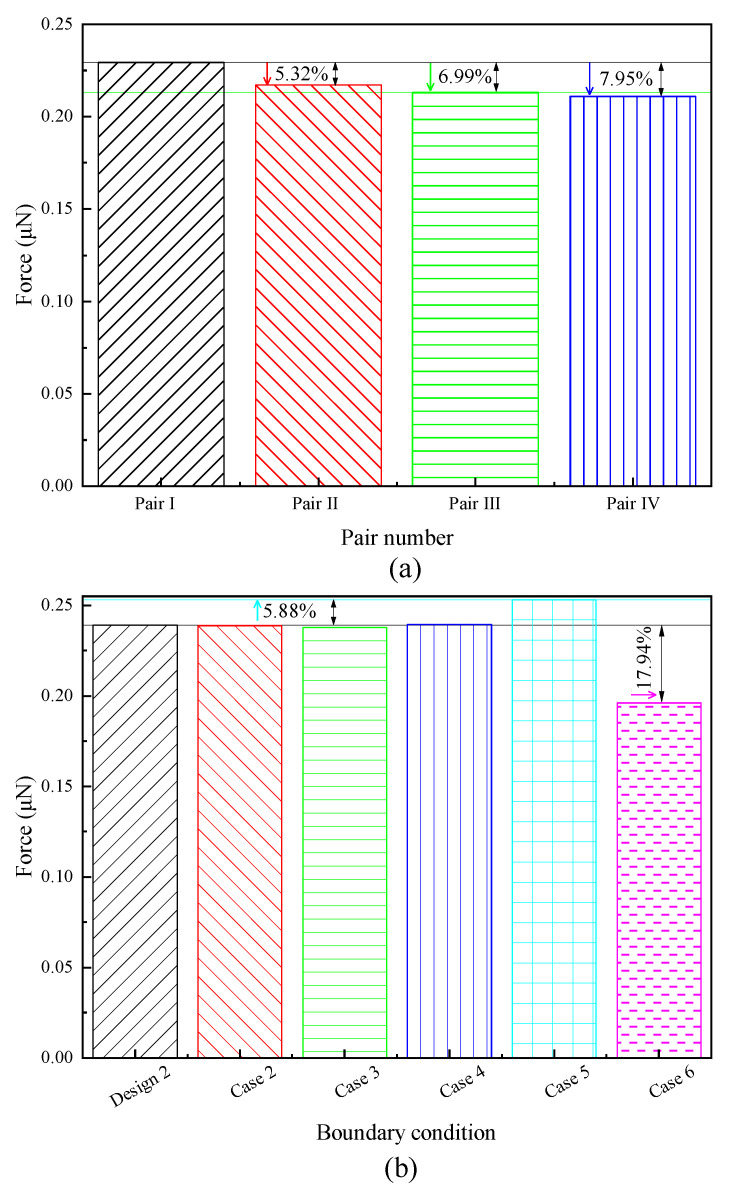
Comparison of the Knudsen forces (one pair) for different boundary conditions and domain sizes. (**a**) Knudsen force for four heater-shuttle pairs, (**b**) Knudsen force for different boundary conditions.

Knudsen forces on the shuttle arms (one pair) for cases 1–6 and design 2 are given in Figure 13. The largest Knudsen force was observed in pair I of case 1, as shown in Figure 13a. Knudsen force decreased gradually from pair I to pair IV. Compared to the value of pair I, the Knudsen forces of pairs II, III, and IV decreased approximately 5.32%, 6.99%, and 7.95%, respectively. It seems that for multiple shuttle-heater pairs, the heater arm of the front pair affects the shuttle arm of the next pair, for example, gas temperature around the shuttle arm would increase and the gas flow patterns change. The coupling effects exist between the shuttle-heater pairs and become weaker toward the right. That is, the independence between pairs is enhanced. This phenomenon can also be found in the previous research [38], but the value of the Knudsen force was slightly different. The reason is that the temperature difference between the shuttle and heater arms was 64 K and the gas pressure was 62 Pa in [38], but in the current work, the temperature difference and pressure were considered as 37.5K and 387 Pa, respectively. Temperature difference and pressure are two crucial factors significantly influencing the value of Knudsen force [2,9,10,11,12,24,25,26,27,28,29,30,31,32,33,34,35,36]. On the other hand, a larger Knudsen force was obtained in the case where only one shuttle-heater pair was simulated, neglecting the coupling effects (Figure 2b). For example, the Knudsen force of the improved design 2 (Figure 2b) increased by approximately 4.3% compared to that of pair I.

As illustrated in Figure 13b, compared to the Knudsen force of the improved design 2, the values of the first three cases 2–4 (Figure 2e–g) were almost equal, but that of case 5 (Figure 2h) was the largest, and increased by approximately 5.88%. The reasons seem to be that (i) the left freestream boundary was closer to the shuttle arm, thus, less gases were blocked by the bottom surface; and (ii) less momentum exchange of gas molecules and shuttle arm surfaces occurred due to a lower temperature of the gas flow from the left freestream boundary, that is, the force acting on the bottom surface was small. Note that according to the analyses of the flow field above, it is emphasized that this largest value is impractical. On the other hand, case 6 (Figure 2i) had the smallest Knudsen force. The smallest value decreased by approximately 17.94% compared to the value of design 2, which can be expected.

## 5. Concluding Remarks

The low-pressure gas sensor based on Knudsen thermal force is revisited in the current work. Three kinds of designs for shuttle arms (Figure 2a–c), gas flow mechanism, coupling effects, and the variation of Knudsen force were investigated. Based on the DSMC method, the results showed that the local mechanism for gas flow was almost similar for all of the cases. The flow field and thermal field showed differences due to different shuttle arm geometric designs. Design 2 returned the highest Knudsen force performance. Compared to the value of the original MIKRA device (Figure 1b), the returned Knudsen force of design 2 was approximately increased to 42.9% with P = 387 Pa. Therefore, design 2 was used to detect the interactions between the shuttle-heater pairs and coupling effects including flow field, thermal characteristics, and Knudsen force.

Coupling effects, similar to those reported in the previous work [38], were found. The effects became weak toward the right, that is, the independence was enhanced. For example, the Knudsen forces on the shuttle arms of pairs II, III, and IV decreased approximately 5.32%, 6.99%, and 7.95%, respectively, when compared to that of pair I. Note that compared to the Knudsen force of pair I, the value of the improved design 2 (Figure 2b) only increased 4.3%, showing that the simulation of one shuttle-heater pair was reasonable to some extent.

On the other hand, regarding cases 2–6 with one shuttle-heater pair for different boundary conditions and simulation domain sizes, it was assumed that case 5 contradicted the physical facts. Case 6 is an ideal situation with countless shuttle-heater pairs. The Knudsen force in cases 5 and 6 increased by 5.88% and decreased by 17.94%, respectively, when compared to the value of the improved design 2 (Figure 2b). Apart from cases 5 and 6, the results of cases 2–4 were almost the same. Therefore, the configuration with the symmetry boundary condition and simulation domain size of 0.36 mm × 0.3 mm (Figure 2g) is recommended for the simulation of gas flow behavior and device performance in future research.

## Figures and Tables

**Table 1:** Temperature of the shuttle and heater arms (average temperature) [25,30].

Pressure P (Pa)	Kn	$T_{c}(K)$	$T_{h}(K)$
62	4.634	304.5	348
155	1.853	304.5	346
387	0.742	304.5	342
966	0.296	303	323
1500	0.184	302	313

**Table 2:** Numerical specification.

Parameter	Value
Maximum cell size Δx=Δy(μm)	2
Particles per cell (PPC)	≥20
Time interval Δt (ns)	1
Number of time interval	$\geq 3\times 10^{6}$
Total number of cell:	
Figure 2a–c	44,520
Figure 2d	124,200
Figure 2e,f	44,520
Figure 2g**–**i	26,460
